# Regulation of lung epithelial cell senescence in smoking-induced COPD/emphysema by microR-125a-5p via Sp1 mediation of SIRT1/HIF-1a

**DOI:** 10.7150/ijbs.65861

**Published:** 2022-01-01

**Authors:** Hao Wu, Huimin Ma, Lumin Wang, Huazhong Zhang, Lu Lu, Tian Xiao, Cheng Cheng, Peiwen Wang, Yi Yang, Meng Wu, Suhua Wang, Jinsong Zhang, Qizhan Liu

**Affiliations:** 1 Department of Emergency, Jiangsu Province Hospital, The First Affiliated Hospital of Nanjing Medical University, Nanjing, 210029, Jiangsu, People's Republic of China.; 2 Center for Global Health, The Key Laboratory of Modern Toxicology, Ministry of Education, School of Public Health, Nanjing Medical University, Nanjing, 211166, Jiangsu, People's Republic of China.; 3 Jiangsu Key Lab of Cancer Biomarkers, Prevention and Treatment, Jiangsu Collaborative Innovation Center for Cancer Personalized Medicine, School of Public Health, Nanjing Medical University, Nanjing, 211166, Jiangsu, People's Republic of China.; 4 Department of Toxicology, School of Public Health, Baotou Medical College, Baotou, 014040, Inner Mongolia, People's Republic of China.

**Keywords:** Smoking, Senescence, Chronic obstructive pulmonary disease, Emphysema, microRNAs

## Abstract

Chronic obstructive pulmonary disease (COPD) affects the health of more than 300 million people worldwide; at present, there is no effective drug to treat COPD. Smoking is the most important risk factor, but the molecular mechanism by which smoking causes the disease is unclear. The senescence of lung epithelial cells is related to development of COPD. Regulation of miRNAs is the main epigenetic mechanism related to aging. β-Galactose staining showed that the lung tissues of smokers have a higher degree of cellular senescence, and the expression of miR-125a-5p is high. This effect is obvious for smokers with COPD/emphysema, and there is a negative correlation between miR-125a-5p levels and values for forced expiratory volume in one second (FEV1)/forced vital capacity (FVC). After Balb/c mice were chronically exposed to various concentrations of cigarette smoke (CS), plethysmography showed that lung function was impaired, lung tissue senescence was increased, and the senescence-associated secretory phenotype (SASP) in bronchoalveolar lavage fluid was increased. For mouse lung epithelial (MLE)-12 cells treated with cigarette smoke extract (CSE), Sp1 and SIRT1 levels were low, HIF-1α acetylation levels were high, and cell senescence and secretion of SASP factors were elevated. Down-regulation of miR-125a-5p or up-regulation of Sp1 reversed these effects. In addition, compared with mice exposed to CS, knockdown of miR-125a-5p reduced lung epithelial cell senescence and COPD/emphysema. Therefore, in smoking-induced COPD, elevated miR-125a-5p participates in the senescence of lung epithelial cells through Sp1/SIRT1/HIF-1α. These findings provide evidence related to the pathogenesis of COPD/emphysema caused by chronic smoking.

## Introduction

Chronic obstructive pulmonary disease (COPD) affects the health of more than 300 million people worldwide. In China, the prevalence of COPD for adults aged 40 and over is 13.7%, and that for adults aged 70 and over is 35.5% 1. Since smoking is the most important risk factor for COPD, and rates of morbidity and mortality are increasing, COPD is a major public health problem 2. Following only ischemic heart disease and stroke, it has become the third leading cause of death in the world and has caused economic and social burdens 3. The clinical characteristics of COPD include expectoration, chronic cough, wheezing, slow onset, and long duration 4. Common treatment strategies include smoking cessation, bronchodilator therapy, supplemental oxygen, pulmonary rehabilitation, and lung transplantation 5. There is no effective way to reverse the decline of lung function; the only way to reduce the frequency and severity of acute exacerbations of COPD is to relieve the symptoms and thereby improve the quality of life of COPD patients. Current approaches focus mainly on symptomatic treatment 6. The two clinical phenotypes of COPD are emphysema and bronchiolitis 7. These phenotypes of COPD have different mechanisms. The purpose of this study was to investigate the pathogenesis in the lungs of COPD patients with an “emphysema phenotype”. This is essential for the discovery of new, disease-specific treatment strategies.

Cellular senescence refers to the state of irreversible and permanent cell cycle arrest, which is the cytological basis of senescence 8. Generally, senescent cells are flat, large, and multinucleated 9. DNA damage response (DDR) is a main feature of senescent cells, and the occurrence of DDR is evident after various stimulations 10. Further, senescent cells use increased activity of β-galactosidase in enlarged lysosomes to catalyze the color reaction of cells at pH 6.0, so that senescent cells can be histologically identified 11. The high expressions of cell cycle regulators such as p16, p53, p21, p15, and p27 lead to irreversible cell cycle arrest 12. The mechanisms related to cellular senescence, which is closely related to COPD, are complicated. COPD is called “accelerated lung senescence disease” 13. In the pathological process of COPD, there is the phenomenon of premature senescence 13.

Cigarette smoke (CS) is an established risk factor for COPD, and the oxidative stress caused by CS is related to various mechanisms that lead to cell senescence and to the senescence-related secretory phenotype (SASP) 14. In epithelial cells and fibroblasts, CS induces the expression of the senescence markers p16, p19, and p21 in the development of emphysema 15. In addition, senescent cells lose their capacity to regenerate, thereby preventing renewal and repair of cells in the lungs and leading, over time, to the development of emphysema and reduced lung function 16. At present, the mechanism for CS-induced senescence of lung epithelial cells is unclear. Therefore, to clarify its relationship with emphysema, we explored the mechanisms involved in CS damage and the senescence of lung epithelial cells.

Abnormal microRNA (miRNA) expression is a characteristic for many human diseases, and miRNAs have a positive or negative role in the formation and progression of diseases 17. They act on the 3'-untranslated regions (UTRs) of mRNAs to regulate the stability and translation of mRNAs encoding target proteins, and negatively regulate gene expression at the post-transcriptional level 18. The regulation of miRNAs is a main senescence-related epigenetic mechanism 7. Certain miRNAs, such as miR-125a-5p, miR-34a-5p, and miR-20a, participate in the senescence process by regulating senescence-related molecules 19, 20. Since, at present, there are few studies on the function of miRNA in the aging process, it is necessary to explore its molecular mechanism.

Specific protein 1 (Sp1) is a ubiquitous, stress-activated transcription factor in the nucleus that regulates genes involved in apoptosis and senescence 21. Its activity is controlled by signal pathways and cell conditions, which affect its interaction with various binding partners, thereby regulating Sp1-dependent transcription 22, 23. The sirtuin (SIRT), or Silent Information Regulator 2 (Sir2), protein has nicotinamide adenine dinucleotide (NAD+)-dependent deacetylase and adenosine diphosphate (ADP)-ribosyl transferase activity. In this family of proteins, SIRT1 has been most extensively investigated 24. SIRT1 acts through deacetylation of its substrates (including the acetylated histones, H4K16 and H3K56) and non-histone targets (p53, FOXO3, and HIF-1α) 25. SIRT1 has biological functions in inflammation, calorie restriction/energy, stress resistance, senescence, endothelial function, and apoptosis/autophagy 26. Sp1 interacts with SIRT1, a downstream target, and enhances the expression of SIRT1 27, 28. The Sp1/SIRT1/HIF-1α signaling pathway is involved in cell senescence. However, the involvement of these pathways in COPD/emphysema induced by CS is unclear.

In the present investigation, we found that miR-125a-5p is highly expressed in lung tissues of smokers and smokers with COPD/emphysema, and the degree of cellular senescence increases with time of exposure. Murine lung epithelial (MLE)-12 cells were exposed to cigarette smoke extract (CSE), and the molecular changes were evaluated. For these cells, high expression of miR-125a-5p enhances activation of the SIRT1/HIF-1α signaling pathway through Sp1 and promotes their senescence. For mice in a COPD/emphysema model that were exposed to CS, knockdown of miR-125a-5p reduced the degree of senescence and emphysema. The results provide a better understanding of the molecular mechanisms of senescence and COPD/emphysema.

## Materials and methods

### Patients and samples

Lung tissue samples were obtained from 60 patients with operable, non-malignant pulmonary nodules recruited from Jiangsu Province Hospital. Their average ages were similar. The informed consent of patients was obtained before the operations, and the project was approved by the Ethics Committee of Jiangsu Province Hospital. The selected patients were divided into three groups based on their smoking history and lung function tests: never smokers, smokers, and smokers with COPD/emphysema. Smokers refer to non-COPD smokers with normal pulmonary function (n=20), smokers with COPD/emphysema were classified by guidelines of the Global Initiative for COPD Prevention and Treatment (GOLD, 2018) 29. A FEV1/FVC (forced expiratory volume in the first second/strength vital capacity) ratio of <70% and clinical diagnosis confirmed COPD patients with the emphysema phenotype (n=20). The surgical patients involved had no underlying lung disease or severe cardiopulmonary disease. The clinical characteristics of the patients are summarized in Table S1.

### Animals and administration of adenovirus

The methods involved in the mouse COPD model have been described previously 30. In short, mice were exposed to CS with 0, 100, 200, or 300 mg/m^3^ total particulate matter (TPM) in a whole-body exposure system (Beijing Huironghe Technology Co., Ltd., China) for a total of 16 weeks. The AAV6-mmu-miR-125a-5p-inhibitor was synthesized by Genechem Technology Co., Ltd. (Genechem, China). The mice were divided into four groups (control, CS, CS+AAV6-mmu-miR-125a-5p-inhibitor, and CS+AAV negative control). The virus titer was 4.2E+12 v.g./ml. After 4 weeks of exposure to CS, adenovirus was administered by intranasal instillation. All animal experiments were approved by the Animal Ethics Committee of Nanjing Medical University and complied with current animal protection and welfare guidelines.

### Lung histopathology and IHC

Lung tissue samples were exposed to fixative, dehydrated with alcohol and xylene, embedded in paraffin, and sliced into sections with a thickness of 4 μm using a paraffin microtome. According to the manufacturer's recommendations, hematoxylin and eosin (H&E) and p21 and p27 (Abcam, China) were used for IHC staining (Solarbio Life Science, China). H-SCOREs ranged from 0 to 300. All staining was assessed by a quantitative imaging method; the percentage of immunostaining and the staining intensity were recorded. The intensity of specific staining was characterized as not present (0), weak but detectable above control (1+), distinct (2+), and very strong (3+). An H-score was calculated using the following formula: H- SCORE=∑ (PI × I) = (percentage of cells of weak intensity × 1) + (percentage of cells of distinct intensity × 2) + (percentage of cells of strong intensity × 3). PI indicated the percentage of positive cells vs. all cells, and I represented the staining intensity.

### Senescence-associated β-galactosidase activity

According to the manufacturer's recommendations, the senescence of cells was assessed by use of senescence-related β-galactosidase (SA-β-gal) staining kits (Beyotime Biotechnology). In short, the cells were washed once with PBS and fixed with β-galactosidase staining fixative solution for 15 min at room temperature. The solution was removed. The cells were washed 3 times with PBS and then incubated with SA-β-gal staining solution at 37 °C overnight. On the second day, the cells were observed under an optical microscope. Cells positive for SA-β-gal (blue staining) were considered as senescent cells, and their percentages were calculated by counting 150 to 200 cells in 3 fields of view.

### Cell culture and treatment

MLE-12 cells were purchased from the Shanghai Institute of Cell Biology, Chinese Academy of Sciences (Shanghai, China). The cells were maintained in RPMI-1640 medium supplemented with 10% FBS, 100 U/mL penicillin, and 100 mg/mL streptomycin (Life Technologies/Gibco, Oshima, New York), in an incubator at 37 °C and 5% CO_2_. Cells were passaged at a ratio of 1:3 every 2 days, and, to induce senescence, were treated with 8% CSE for 0, 2, 4, or 8 generations.

### Preparation of CSE

CSE was prepared as previously reported 31. Briefly, the smoke of a 3R4F Research Cigarette (University of Kentucky, USA) was bubbled into a flask containing 10 mL of warm (37 °C) RPMI-1640 medium by use of a vacuum pump at a constant speed. (Each cigarette was smoked for 5 min.) The CSE solution was adjusted to pH 7.4 and then sterilized by filtration through a 0.22-µm pore filter (Schleicher & Schuell GmbH, Dassel, Germany). For quality control, the solution was standardized by monitoring the absorbance at 320 nm (A320) and 540 nm (A540). CSE quality was accepted if ΔOD (A320-A540) was between 0.9 and 1.2. The resulting solution was regarded as 100 % CSE and, within 1 h, was diluted with medium for use in experiments.

### Lung function measurement

Plethysmography (Buxco Electronics Ltd., USA) measures airway hyper-responsiveness (AHR) for mice to assess lung function 30. We placed 4 mice into individual, airtight boxes; turned on the oxygen valve; and allowed the mice to adapt to the arrangement for 5 minutes. We added acetylcholine (0, 12.5, 25, or 50 mg/ml) for aerosol inhalation for 2 minutes. The reaction and recording time was 5 minutes, and the recovery time was 2 minutes. FinePoint software (Buxco Electronics Ltd., USA) was used to record relevant parameters. Penh is a unitless value used to reflect the extent of airway resistance.

### Western blots

By use of 10% sodium dodecyl sulfate-polyacrylamide gel electrophoresis, equal amounts (80 μg) of protein were separated and were transferred to nitrocellulose membranes (Millipore, Billerica, MA). Membranes were incubated with a 1:1000 dilution of antibodies for p21, p27, Sp1, SIRT1, acetyl-HIF-1α, HIF-1α, or GAPDH (Abcam, China) overnight at 4 °C. The preparations were subjected to additional incubation with a 1:10000 dilution of an anti-immunoglobin horseradish peroxidase-linked antibody (Beijing Zhongshan Jinqiao Biological Technology Co., Beijing, China) at room temperature for 1 hr. Finally, proteins were detected by ECL reagents (BIO-RAD, USA), and densities of bands were quantified by Image J software.

### Quantitative real-time PCR

According to the manufacturer's recommendations, 1 μg of total RNA was reverse-transcribed into cDNA using HiScript II Q Select RT Supermix. The amplification step was completed by use of Power SYBR Green Master Mix (Vazyme Biotech, China) and a LightCycler 96 instrument (Roche, Swiss). The 2^-ΔΔCT^ method was used to calculate the results. The primer sequences are in Table S2.

### Enzyme-linked immunosorbent assay

According to the manufacturer's recommendations, we used ELISA kits (BIOHJ.com, China) to measure the concentrations of IL-6, IL-8, and CCL2. The absorbance (OD value) was determined with a microplate reader at a wavelength of 450 nm. The concentrations of IL-6, IL-8, and CCL2 in the samples were calculated from a standard curve.

### Chromatin immunoprecipitation (ChIP) assays

Chromatin immunoprecipitation was performed using SimpleChIP® Enzyme Chromatin IP Kits (Cell Signaling Technology) according to the manufacturer's instructions. Cells were immunoprecipitated with Sp1 or SIRT1 antibodies or normal IgG (Cell Signaling Technology) and then amplified by PCR. The primers for the SIRT1 promoter are listed in Table S2.

### Luciferase reporter assays

The binding of miR-125a-5p to Sp1 was determined by a luciferase reporter gene assay. The wild-type or mutant Sp1 3'-UTR was cloned into the psiCHECK2 vector (GeneRay, China) and co-transfected into MLE-12 cells with an miR-125a-5p mimic or con mimic. The luciferase activity was measured with dual luciferase reporter kits (Beyotime, China).

### Statistical analysis

All experiments were repeated three times independently. Values were expressed as means ± SD. Comparison of means between multiple groups was performed by one-way analysis of variance (ANOVA), and multiple range minimum significant difference (LSD) was used for comparison between groups. P < 0.05 was considered statistically significant. Data were analyzed by use of SPSS 18.0.

## Results

### Smoking increases senescence-related indicators in human lung tissue and levels of miR-125a-5p during induction of COPD/emphysema

**Figure 1:**
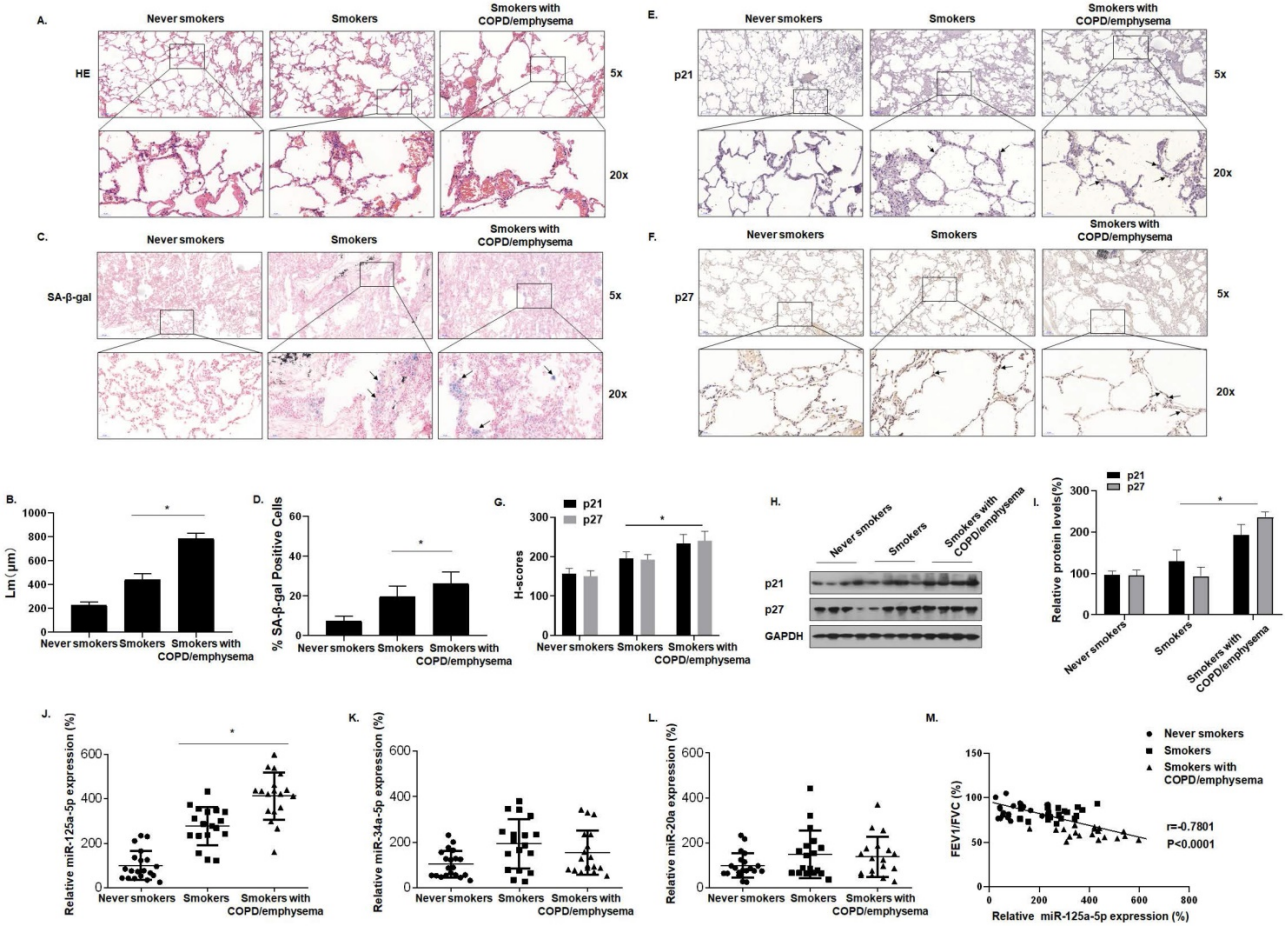
** Smoking increases senescence-related indicators in human lung tissue and levels of miR-125a-5p during induction of COPD/emphysema.** Densities of bands were quantified by Image J software. GAPDH levels, measured in parallel, served as controls. Lung tissues were collected from never smokers (n = 20), smokers (n = 20), and smokers with COPD/emphysema (n = 20). **(A)** Representative images of H&E staining of lung tissues and **(B)** mean linear intercept (µM) measurements (n = 6). **(C)** Photographs of senescence-associated β-galactosidase (SA-β-gal) staining and **(D)** the percentages of SA-β-gal positive cells (n = 6, arrow: stained positive cells). **(E and F)** Representative immunostaining images and **(G)** the levels of p21 and p27 in lung tissues were determined by IHC (n = 6, arrow: stained positive cells). **(H)** Western blots were performed, and **(I)** relative protein levels of p21 and p27 in the lung tissues were determined (n = 3). The levels of miR-125a-5p **(J)**, miR-34a-5p **(K)**, and miR-20a **(L)** in the lung tissues were determined by qRT-PCR. **(M)** The correlation between FEV1/FVC ratios and miR-125a-5p levels was determined. Values were expressed as means ± SD. ^*^ p < 0.05 compared with controls.

Table S1 shows the clinical data for the participants. Compared with never smokers, smokers and smokers with COPD/emphysema had different degrees of alveolar expansion as determined by the H&E staining of lung tissue. Further, the alveolar compartments were broken, and the expanded alveoli had fused into larger cyst cavities. This situation was more pronounced for smokers with COPD/emphysema (Fig. 1A and B). Senescence β-galactosidase (SA-β-gal), which is overexpressed in senescent cells, is widely used as a marker of cell senescence. Cells staining blue are considered to be senescent. SA-β-gal staining was elevated in lung tissue of smokers with COPD/emphysema (Fig. 1C and D). Immunohistochemical (IHC) analysis showed that, in the lungs of never-smokers, there was minimal staining of p21 and p27, proteins that regulate the cell cycle and which serve as markers for cellular senescence. Although their expression in lung alveolar epithelial cells of never-smokers was relatively low, and expression in smokers was moderately higher, these cells of smokers with COPD/emphysema showed high expression (Fig. 1E, F, and G). As determined by Western blots, the lung tissues of smokers with COPD/emphysema, relative to tissues of never smokers and smokers, showed high expression of p21 and p27 proteins (Fig. 1H and I).

We also evaluated the expression of senescence-related miRNAs in lung tissues. For the three groups, miR-125a-5p, miR-34a-5p, and miR-20a, measured by qRT-PCR, showed that the expression of miR-125a-5p alone was higher in the three groups (Fig. 1J, K and L). For these subjects, the FEV1/FVC ratios and miR-125a-5p expression were included in correlation analyses. FEV1/FVC ratios negatively correlated with miR-125a-5p expression (Fig. 1M). These results show that senescence of lung epithelial cells is likely related to the development of COPD/emphysema and that, during the induction of COPD/emphysema, smoking elevates senescence-related indicators and miR-125a-5p levels in lung tissue.

### Senescence-related indicators and miR-125a-5p are up-regulated in the lung tissues of mice by long-term exposure to CS

**Figure 2:**
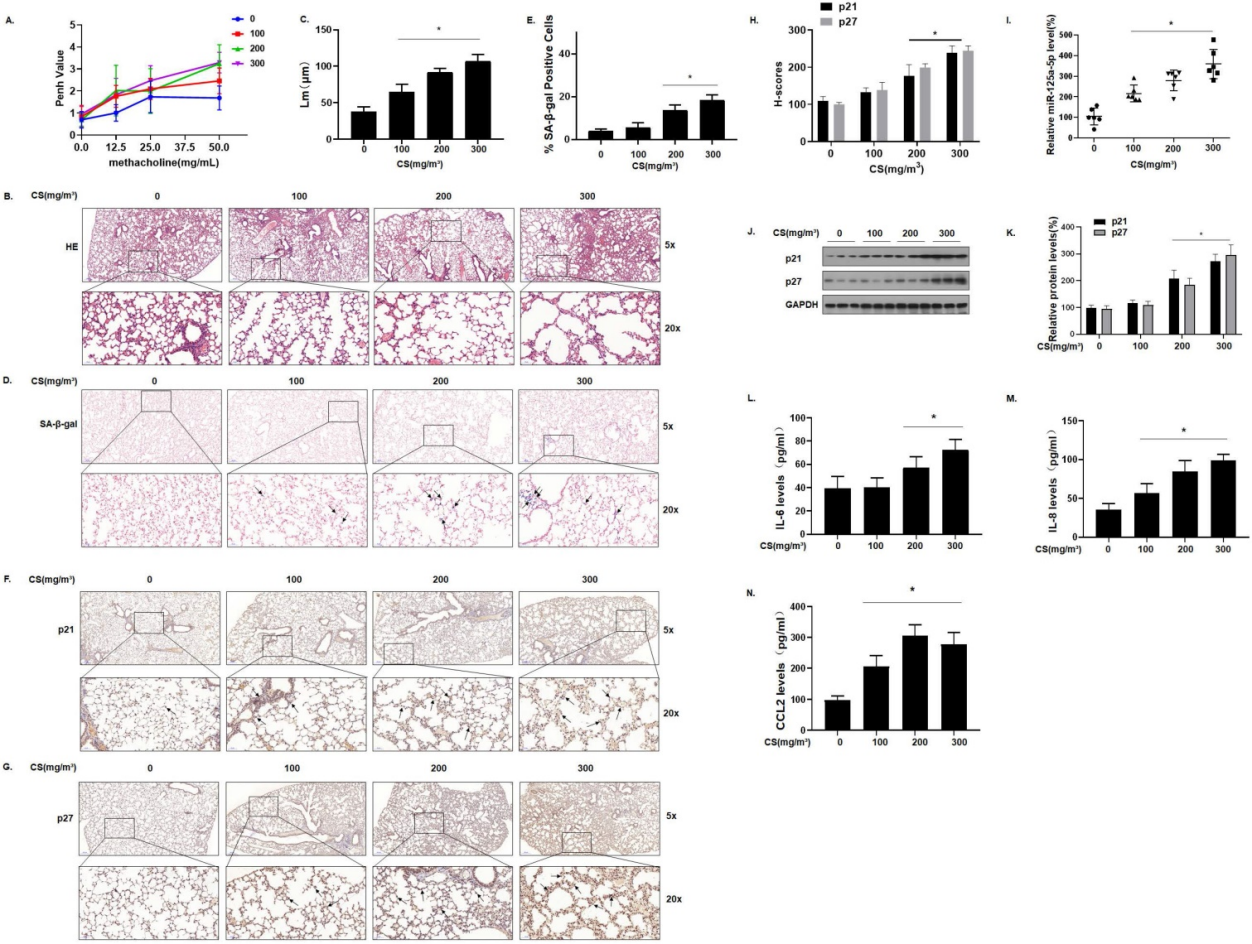
** Senescence-related indicators and miR-125a-5p are up-regulated in the lung tissues of mice by long-term exposure to CS.** Densities of bands were quantified by Image J software. GAPDH levels, measured in parallel, served as controls. Male BALB/c mice at 6-8 weeks of age were exposed to 0, 100, 200, or 300 mg/m^3^ TPM CS for 16 weeks. **(A)** Penh values were measured by use of whole-body plethysmography (n = 6). **(B)** Representative images of H&E staining in lung tissue of mice and **(C)** mean linear intercept (µM) measurements (n = 6). **(D)** Photographs of senescence-associated β-galactosidase (SA-β-gal) staining and **(E)** percentages of SA-β-gal positive cells (n = 6, arrow: stained positive cells). **(F and G)** Representative immunostaining images and **(H)** the levels of p21 and p27 in lung tissues of mice were determined by IHC (n = 6, arrow: stained positive cells). **(I)** Levels of miR-125a-5p in the lung tissues were determined by qRT-PCR (n = 6). **(J)** Western blots were performed, and **(K)** relative protein levels of p21 and p27 in the lung tissues were determined (n = 3). The levels of IL-6 **(L)**, IL-8 **(M)**, and CCL2 **(N)** in mouse BALF were assessed by ELISA (n = 6). Values were expressed as means ± SD. ^*^ p < 0.05 compared with controls.

To determine the relationship between cellular senescence and COPD/emphysema, a COPD/emphysema model of mice exposed to CS was established as described previously 30. Balb/c mice were placed in a whole-body system for exposure to CS at concentrations of 0, 100, 200, or 300 mg/m^3^ total particulate matter (TPM). After 16 weeks, the methacholine challenge test showed that CS exposure affected the lung function of mice, and airway hyper-responsiveness (AHR) was elevated in a concentration-dependent manner (Fig. 2A). The lung tissues of mice were obtained for experiments. H&E staining showed that, compared with the control group, the lungs of the mice in other groups had various degrees of pathological manifestations of emphysema, as determined by more extensive alveolar spacing and ruptures of the alveolar walls (Fig. 2B and C). Further, we evaluated the senescence of cells in the lung tissues of each group of mice. The results of SA-β-gal staining of lung tissue showed that, with higher CS exposure, the percentages of SA-β-gal staining cells were higher (Fig. 2D and E).

We measured the expression of the senescence markers p21 and p27 in the lung tissues of mice; IHC results showed that p21 and p27 stained more extensively in the lung tissues of mice in the high-exposure group, indicating that their expression levels were higher (Fig. 2F, G and H). The qRT-PCR results showed that, compared with the control group, the levels of miR-125a-5p increased in other groups in a concentration-dependent manner (Fig. 2I). Measurement of the protein levels showed that, compared with the control group, the expression of p21 and p27 in lung tissues was increased in a concentration-dependent manner (Fig. 2J and K). Cell senescence is accompanied by appearance of the senescence-associated secretory phenotype (SASP); secreted SASP factors amplify harmful effects and cause damage to neighboring cells 32. The main components of SASP, IL-6, IL-8, and CCL2 were measured by ELISA. Compared with the control group, the levels of these components in mouse bronchoalveolar lavage fluid (BALF) were elevated (Fig. 2L, M, and N), suggesting that long-term exposure to CS causes up-regulation of miR-125a-5p and senescence-related indicators and in the lung tissues of COPD/emphysema mice.

### CSE exposure leads to senescence and increases of levels of SASP factors in MLE-12 cells

**Figure 3:**
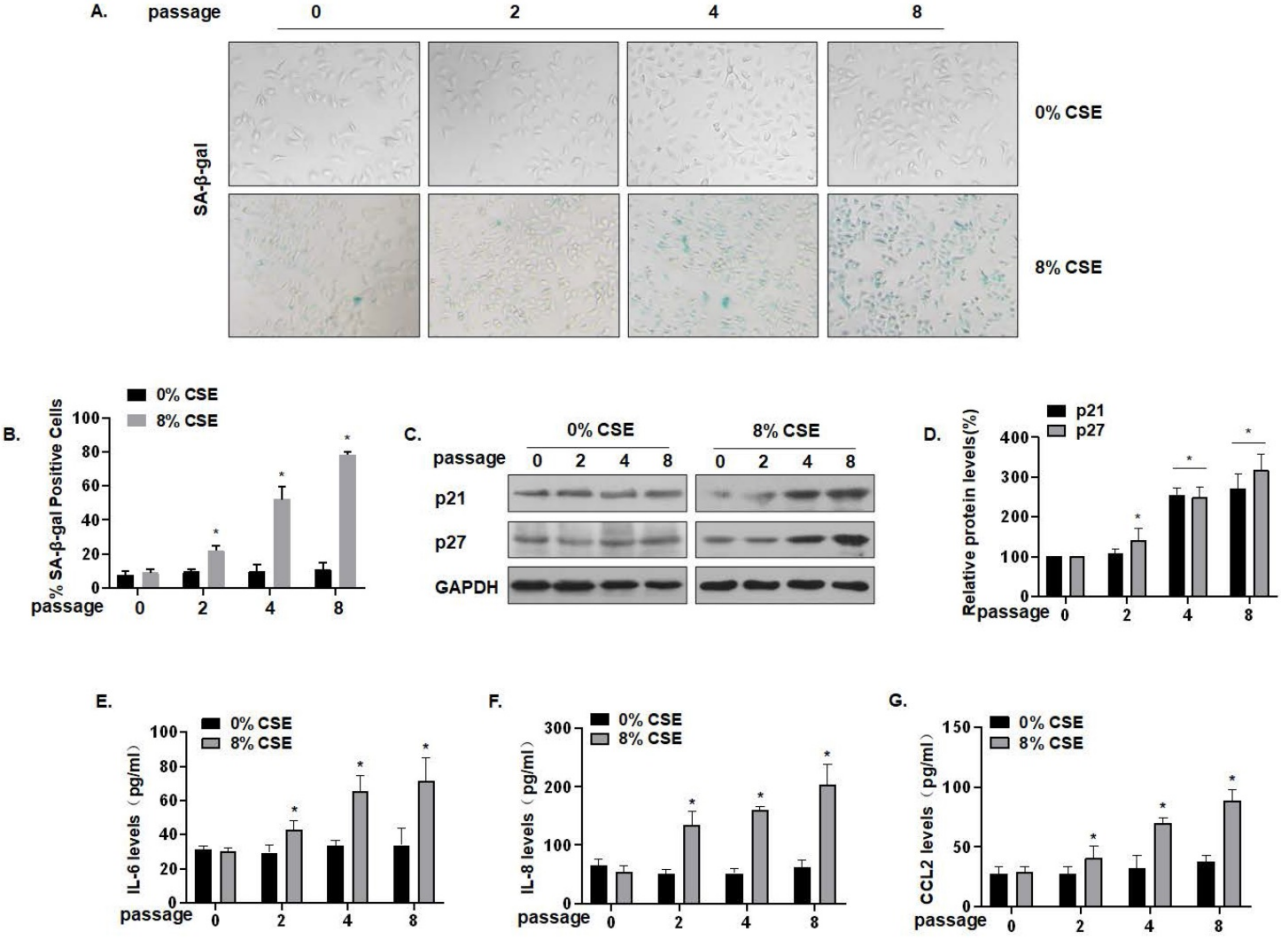
** CSE exposure leads to senescence and increases of levels of SASP factors in MLE-12 cells.** Densities of bands were quantified by Image J software. GAPDH levels, measured in parallel, served as controls. MLE-12 cells were treated with 0 or 8% CSE for 0, 2, 4, or 8 passages. **(A)** Photographs of cells with senescence-associated β-galactosidase (SA-β-gal) staining and **(B)** the percentage of SA-β-gal positive cells (n = 3). **(C)** Western blots were performed, and **(D)** relative protein levels of p21 and p27 in MLE-12 cells were determined (n = 3). The levels of IL-6 **(E)** IL-8 **(F)**, and CCL2 **(G)** in MLE-12 cells were assessed by ELISA (n = 3). Values were expressed as means ± SD. ^*^ p < 0.05 compared with controls.

To further characterize the effect of CS on lung epithelial cells, we established an *in vitro* cell senescence model. Specifically, 0, 2, 4, or 8 passages of MLE-12 cells were exposed to 0 or 8% CSE. SA-β-gal staining showed that, compared with the control of the same generation, the number of senescent cells was somewhat increased after treatment with 8% CSE for 4 generations, and that the number of senescent cells was substantially increased after treatment for 8 generations. The cells became larger and showed a flat, irregular shape, which is typical of senescent cells (Fig. 3A) and more cells became positive for SA-β-gal (Fig. 3B). Compared with the control group of the same passage, the protein levels of MLE-12 cell senescence markers, p21 and p27, in the CSE treatment groups were up-regulated (Fig. 3C and D). In addition, the secretion of SASP factors into the cell media was measured by ELISA. Compared with the control group of the same generation, the levels of IL-6, IL-8, and CCL2 secreted by MLE-12 cells in the 8% CSE treatment group were increased in a time-dependent manner (Fig. 3E, F, and G). Therefore, these results show that CSE exposure induces senescence in MLE-12 cells and elevates levels of SASP factors.

### miR-125a-5p via Sp1 regulates SIRT1 and acetylated HIF-1α in CSE-treated MLE-12 cells

**Figure 4:**
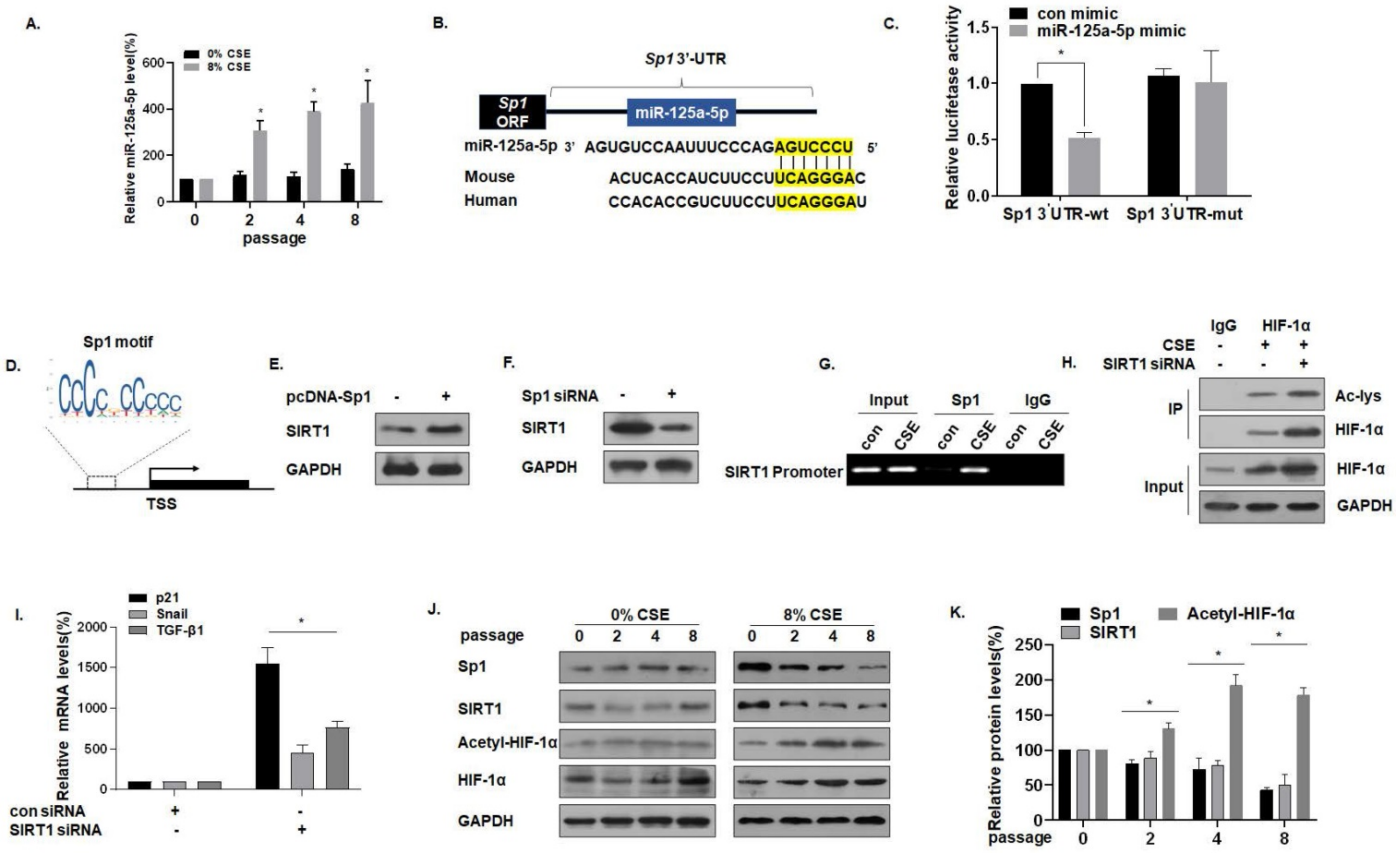
** miR-125a-5p via Sp1 regulates SIRT1 and acetylated HIF-1α in CSE-treated MLE-12 cells.** Densities of bands were quantified by Image J software. GAPDH levels, measured in parallel, served as controls. MLE-12 cells were treated with 0 or 8% CSE for 0, 2, 4 or 8 passages. MLE-12 cells were transfected with psiCHECK2-Sp1 3'-UTR-wt or psiCHECK2-Sp1 3'-UTR-mut for 24 h, then transfected with an miR-125a-5p mimic (50 nM). **(A)** The levels of miR-125a-5p in MLE-12 cells were measured by qRT-PCR (n = 3). ^*^ p < 0.05 compared with controls. **(B)** The predicted complementary binding sites within miR-125a-5p and Sp1. **(C)** Luciferase reporter assays were performed with MLE-12 cells (n = 3). * p < 0.05 different from MLE-12 cells co-transfected with a con mimic. **(D)** Binding site of the Sp1 sequence and the SIRT1 promoter region. **(E)** Western blots were performed and **(F)** relative protein levels of SIRT1 were assessed. **(G)** Endogenous binding of Sp1 to the SIRT1 gene promoter was determined for the chromatin immunoprecipitated by an anti-Sp1 antibody or an anti-IgG antibody (as a control). **(H)** The same amount of protein was immunoprecipitated, and then immunoblotting was performed with antibodies against HIF-1α or acetyllysine. **(I)** The mRNA levels of TGF-β1, Snail, and p21 were determined by qRT-PCR (n = 3). **(J)** Western blots were performed, and (K) the relative protein levels of Sp1, SIRT1, Acetyl-HIF-1α, and HIF-1α in MLE-12 cells were determined (n = 3). Values were expressed as means ± SD. ^*^ p < 0.05 compared with controls.

For MLE-12 cells in the process of developing senescence, the expression of miR-125a-5p gradually increased (Fig. 4A). Sequence analysis shows that miR-125a-5p is highly conserved in Homo sapiens and in mice. Data from the bioinformatics website TargetScan (http://www.targrtscan.org/) was used to predict that miR-125a-5p has a binding site in the 3'-UTR region of Sp1 mRNA (Fig. 4B). The results of luciferase reporter gene assays revealed that the fluorescence intensity of MLE-12 cells co-transfected with Sp1-wt and an miR-125a-5p mimic was lower, showing miR-125a-5p and Sp1 targeted binding (Fig. 4C). *Sp1* and *SIRT1* are genes involved in the aging process 33, and SIRT1 is the downstream target of Sp1. Through the JASPAR database (http://jaspar.genereg.net/), the Sp1 binding site was identified in the SIRT1 promoter region (Fig. 4D). In exploring the regulatory function of Sp1 on SIRT1, we observed that the overexpression of Sp1 increased the levels of SIRT1 (Fig. 4E), but knockdown of Sp1 reduced the levels of SIRT1 (Fig. 4F). CHIP experiments confirmed that, in MLE-12 cells, Sp1 binds to the SIRT1 promoter (Fig. 4G), which is consistent with results of a previous study 28. SIRT1 commonly regulates protein activity through the deacetylation of lysine residues; SIRT1 deacetylates HIF-1α and inhibits its transcriptional activity on downstream target genes 34. To test this hypothesis for MLE-12 cells, they were treated with 0% or 8% CSE for 8 generations, then transfected with SIRT1 siRNA. The same amount of protein was immunoprecipitated with HIF-1α antibody, and Western blot analysis of immunoprecipitates with acetyllysine antibody was performed. In the cells, SIRT1 was depleted, and the acetylation level of HIF-1α increased (Fig. 4H). Further, in order to study the increase of HIF-1α acetylation level, the activity of HIF-1α on downstream target genes, mRNA levels of TGF-β1, Snail, and p21 were measured by qRT-PCR. mRNA levels of TGF -β1, Snail, and p21 were higher after knocking down SIRT1, indicating that acetylation of HIF-1α lysine was related to the higher HIF-1α transcription activity (Fig. 4I). Compared with the control of the same passage, with the increase of CSE treatment passages, the levels of Sp1 and SIRT1 decreased, and the levels of HIF-1α acetylation increased (Fig. 4J and K). These results indicate that miR-125a-5p via Sp1 regulates SIRT1 and acetylated HIF-1α in CSE-treated MLE-12 cells.

### miR-125a-5p is involved in CSE-mediated MLE-12 cells senescence and secretion of SASP factors

**Figure 5:**
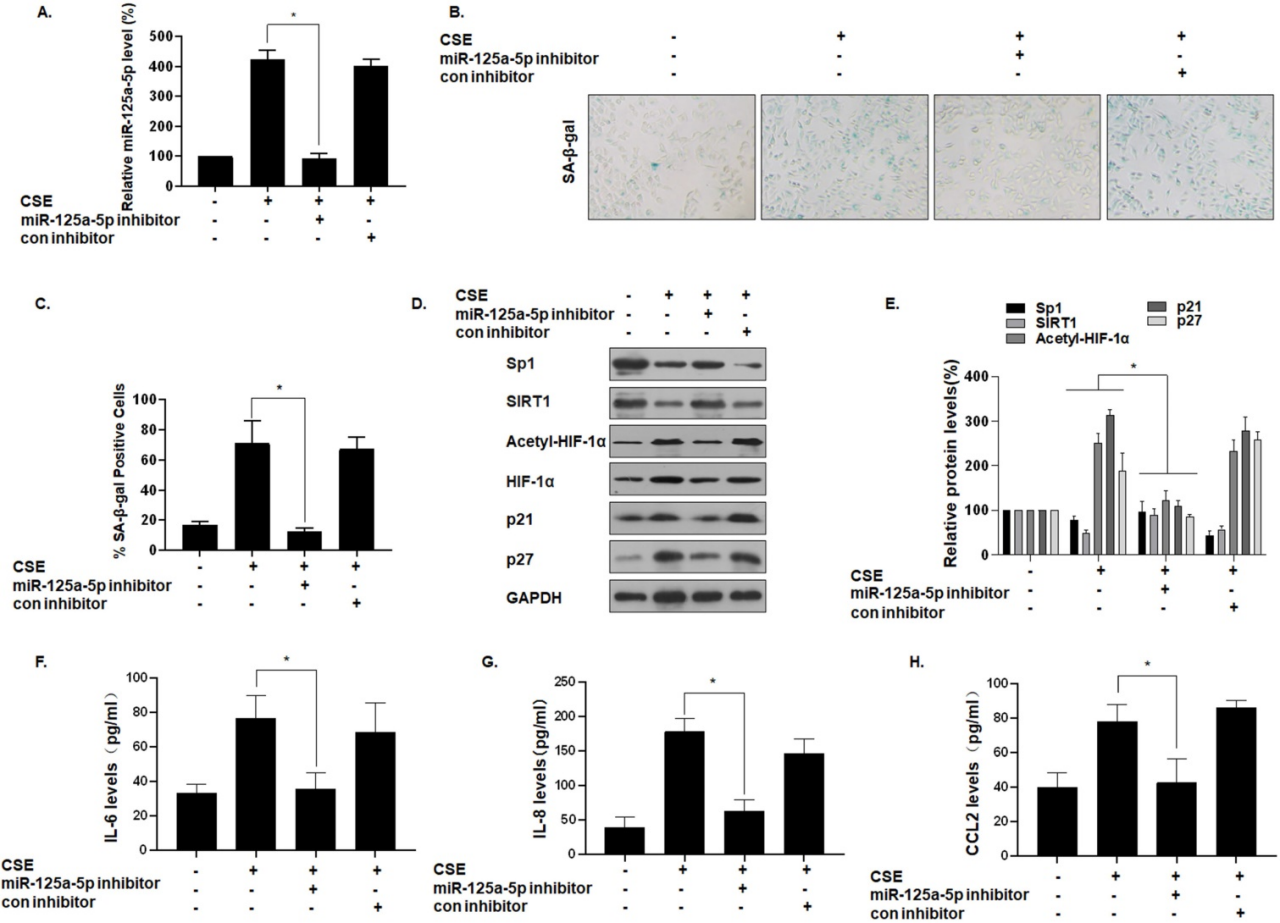
** miR-125a-5p is involved in CSE-mediated MLE-12 cell senescence and secretion of SASP factors.** Densities of bands were quantified by Image J software. GAPDH levels, measured in parallel, served as controls. MLE-12 cells were exposed to 0 or 8% CSE for 48 h after cells were transfected with an miR-125a-5p inhibitor or a con inhibitor for 24 h. **(A)** The levels of miR-125a-5p in MLE-12 cells were measured by qRT-PCR (n = 3). **(B)** Photographs of senescence-associated β-galactosidase (SA-β-gal) staining and **(C)** the percentage of SA-β-gal positive cells (n = 3). **(D)** Western blots were performed, and **(E)** the relative protein levels of Sp1, SIRT1, acetyl-HIF-1α, HIF-1α, p21, and p27 in MLE-12 cells were determined (n = 3). The levels of IL-6 **(F)**, IL-8 **(G)** and CCL2 **(H)** in MLE-12 cells were assessed by ELISA (n = 3). Values were expressed as means ± SD. ^*^ p < 0.05 compared with individual CSE treatment groups.

To determine the function of miR-125a-5p on MLE-12 cell senescence, we reduced its expression by transfecting cells with an miR-125a-5p inhibitor (Fig. 5A). Compared with the group of CSE treatment only, after miR-125a-5p was knocked down, the number of SA-β-gal-positive cells decreased (Fig. 5B and C). In addition, Western blot analysis showed that, compared with the CSE treatment group, the group with knockdown of miR-125a-5p had elevated Sp1 and SIRT1 expression, decreased HIF-1α acetylation levels, and decreased cell senescence markers p21 and p27 (Fig. 5D and E). ELISA showed that transfection of an miR-125a-5p inhibitor reduced the secretion of IL-6, IL-8, and CCL2, the main components of SASP in MLE-12 cells (Fig. 5F, G, and H). Thus, inhibition of miR-125a-5p reduces the senescence of MLE-12 cells, and miR-125a-5p is a positive regulator of MLE-12 cell senescence. These data confirm that miR-125a-5p is involved in CSE-mediated MLE-12 cell senescence and in levels of secretion of SASP factors. Similarly, the Sp1 overexpression plasmid was transfected to inhibit the senescence of MLE-12 cell (Fig. S1).

### miR-125a-5p, via Sp1 regulation of SIRT1/HIF-1α, is involved in the senescence and secretion of SASP factors in CSE-treated MLE-12 cells

**Figure 6:**
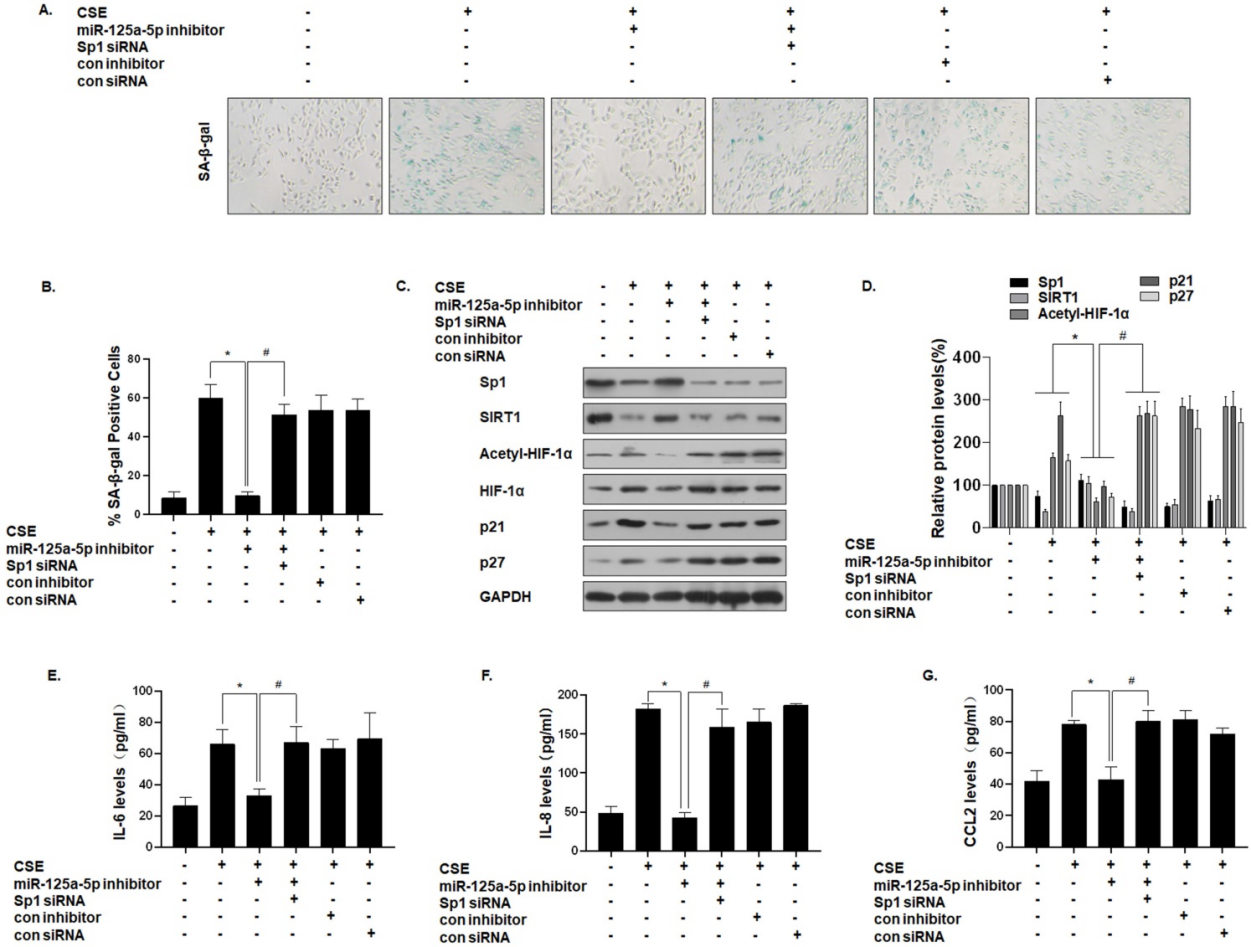
** miR-125a-5p, via Sp1 regulation of SIRT1/HIF-1α, is involved in the senescence and secretion of SASP factors in CSE-treated MLE-12 cells.** Densities of bands were quantified by Image J software. GAPDH levels, measured in parallel, served as controls. MLE-12 cells were exposed to 0 or 8% CSE for 48 h after cells were transfected with a pcDNA-Sp1 or a pcDNA-con. MLE-12 cells were exposed to 0 or 8% CSE for 48 h after they were co-transfected with Sp1 siRNA or con siRNA and an miR-125a-5p inhibitor or a con inhibitor for 24 h. **(A)** Photographs of senescence associated β-galactosidase (SA-β-gal) staining and **(B)** the percentage of SA-β-gal positive cells (n = 3). **(C)** Western blots were performed, and **(D)** the relative protein levels of Sp1, SIRT1, acetyl-HIF-1α, HIF-1α, p21, and p27 in MLE-12 cells were determined (n = 3). The levels of IL-6 **(E)**, IL-8 **(F)** and CCL2 **(G)** in MLE-12 cells were assessed by ELISA (n = 3). Values were expressed as means ± SD. ^*^ p < 0.05 compared with individual CSE treatment groups, ^#^ p < 0.05 compared with CSE-treated MLE-12 cells transfected with the miR-125a-5p inhibitor alone.

To further establish that miR-125a-5p mediates the effect of Sp1 on MLE-12 cell senescence, we down-regulated the expression of miR-125a-5p and then down-regulated the expression of Sp1. SA-β-gal staining showed that the senescence level of cells co-transfected with the miR-125a-5p inhibitor and Sp1 siRNA was restored (Fig. 6A and B). Western blot analysis showed that, compared with the CSE treatment group, the expressions of Sp1 and SIRT1 in the cells transfected with the miR-125a-5p inhibitor increased, the level of HIF-1α acetylation decreased, and the cell senescence markers p21 and p27 were down-regulated. However, Sp1 knockdown eliminated the inhibitory effect of the miR-125a-5p inhibitor on MLE-12 cell senescence-related proteins and pathway proteins (Fig. 6C and D). Similarly, ELISA analysis showed that, compared with the CSE treatment group, transfection of MLE-12 cells with the miR-125a-5p inhibitor blocked the secretion of IL-6, IL-8, and CCL2 (Fig. 6E, F, and G). Next, on the basis of down-regulating the expression of miR-125a-5p, the expression of Sp1 was further down-regulated, and the secretion levels of IL-6, IL-8, and CCL2 were restored. These results indicate that miR-125a-5p regulates SIRT1/HIF-1α through Sp1 to participate in CSE-mediated MLE-12 cell senescence and SASP factor secretion.

### Inhibition of miR-125a-5p prevents CS-induced senescence and COPD/emphysema in mice by blocking regulation of the Sp1/SIRT1/HIF-1α signaling pathway

**Figure 7:**
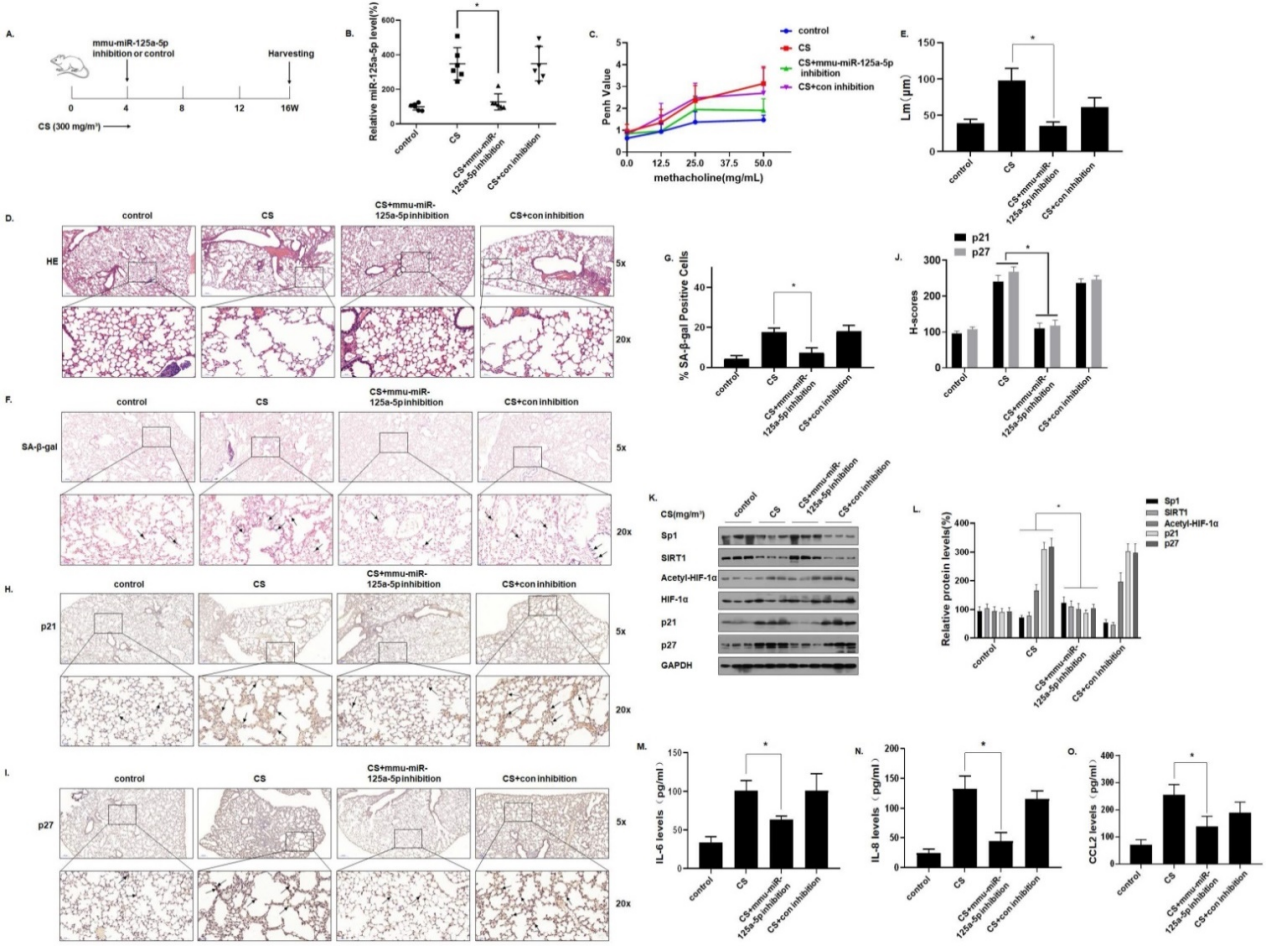
** Inhibition of miR-125a-5p prevents CS-induced senescence and COPD/emphysema in mice by blocking regulation of the Sp1/SIRT1/HIF-1α signaling pathway.** Densities of bands were quantified by Image J software. GAPDH levels, measured in parallel, served as controls. After mice were exposed to 0 or 300 mg/m^3^ TPM of CS for 4 weeks and instilled through the nose with an adeno-associated virus (AAV)6-mmu-miR-125a-5p-inhibitor or an AAV6-mmu-control-inhibitor, they were sacrificed 16 weeks later. **(A)** Schematic diagram of the experimental design and collection. **(B)** The levels of miR-125a-5p in the lung tissues were determined by qRT-PCR (n = 6). **(C)** Penh values were measured by use of whole-body plethysmography (n = 6). **(D)** Representative images of H&E of the lung tissues and **(E)** mean linear intercept (µM) measurements (n = 6). **(F)** Photographs of senescence associated β-galactosidase (SA-β-gal) staining and **(G)** the percentage of SA-β-gal positive cells (n = 6, arrow: stained positive cells). **(H and I)** Representative immunostaining images and **(J)** the levels of p21 and p27 in lung tissues of mice were determined by IHC (n = 6, arrows: stained positive cells). **(K)** Western blots were performed, and **(L)** relative protein levels of p21 and p27 in the lung tissues were determined (n = 3). The levels of IL-6 **(M)**, IL-8 **(N)** and CCL2 **(O)** in mouse BALF were assessed by ELISA (n = 6). Values were expressed as means ± SD. ^*^ p < 0.05 compared with controls.

To verify the function of miR-125a-5p in CS-induced senescence of mice lung cells, an *in vivo* intervention was accomplished. In short, mice were exposed to 0 or 300 mg/m^3^ TPM of CS for 4 weeks and instilled through the nose with an adeno-associated virus (AAV)6-mmu-miR-125a-5p-inhibitor or an AAV6-mmu-control-inhibitor. The mice were sacrificed 16 weeks later (Fig. 7A). After the levels of miR-125a-5p in the lung tissues were determined by qRT-PCR, treatment with the virus (AAV)6-mmu-miR-125a-5p-inhibitor prevented the CS-induced increases of miR-125a-5p levels in lung tissues, which indicated that the intervention was efficient (Fig. 7B). The methacholine challenge test showed that, compared with the CS-exposed group, the lung function of mice was restored when the miR-125a-5p levels were down-regulated (Fig. 7C). H&E staining showed that after down-regulating the level of miR-125a-5p, the pathological manifestations of emphysema in the lungs of mice were reduced (Fig. 7D and E). In addition, the results of SA-β-gal staining of lung tissue showed that, compared with the CS exposure group, after down-regulating the level of miR-125a-5p, the percentage of SA-β-gal staining cells in the lung tissue was lower (Fig. 7F and G). Further, the results of IHC showed that p21 and p27 stained more deeply in the lung tissues of the CS-exposed mice. After down-regulating the level of miR-125a-5p, the expressions of p21 and p27 in the lung tissue was lower (Fig. 7H, I, and J). After down-regulation of miR-125a-5p levels, the expressions of Sp1 and SIRT1 in lung tissue increased, HIF-1α acetylation levels decreased, and p21 and p27 were down-regulated (Fig. 7K and L). ELISA results showed that, compared with the CS-exposed group, after down-regulating the level of miR-125a-5p, the levels of the main components of SASP in mouse BALF, IL-6, IL-8, and CCL2, were lower (Fig. 7M, N, and O). These results show that inhibition of miR-125a-5p prevents CS-induced COPD/emphysema by blocking regulation of Sp1/SIRT1/HIF-1α signaling pathway.

## Discussion

COPD is a burden on global health 35. Although clinical treatment has made progress, there is currently no drug that reduces the disease progression or mortality of COPD 13. Cell senescence is related to the pathogenesis of COPD and is a phenotype for COPD patients. It affects the progression, severity, and prognosis of COPD 36. Oxidative stress caused by exposure to CS may be a cause of COPD senescence 37. Exposure to CS increases the aging markers of respiratory epithelial cells 38. Aged alveolar epithelial cells and endothelial cells accumulate in the lungs of COPD patients 11. In the present study, compared with non-smokers, SA-β-gal staining and senescence-related proteins (including p21 and p27) were found in lung tissues of normal smokers and COPD (smoker) patients compared with non-smokers. This finding was more pronounced for COPD (smoker) patients. To determine the relationship between cellular senescence and COPD/emphysema, we established a COPD/emphysema model for mice exposed to CS. As CS exposure increased, the percentage of SA-β-gal staining cells in lung tissue increased, and the levels of p21 and p27 in lung tissues increased, accompanied by an increase in secretion of SASP factors. Overall, these data indicate that COPD/emphysema is associated with CS-induced senescence of lung epithelial cells.

Cellular senescence is a biological process that occurs in response to a variety of stresses; it is a characteristic of a numerous physiological and pathological processes. Oxidative stress, oncogene activation, chromatin modification, and other forms of stress may cause cell senescence 39. A prominent feature of aging is cell cycle arrest, which exhibits the upregulation of genes that inhibit the cell cycle, such as *p53* (also known as *Trp53* or *TP53*), *p21* (*Cdkn1a*), *p16*, *INK4A*, and *p19Arf*, blocking the cell cycle and establishing an irreversible, stagnant state^40^. In senescent cells, the lysosomal hydrolase, β-galactosidase, which is called “senescence-related β-galactosidase” is expressed 11. In terms of cell morphology, senescent cells become larger, flattened, and vacuous; they also exhibit a complex SASP 41.

Acting as inducers of senescence are various miRNAs, called senescence-related miRNAs (SA-miRNA or SA-miRs). miRNAs negatively regulate gene expression and control cellular senescence 42. miRNAs that target the p53/p21 and p16/pRb senescence pathways and are associated with the SASP pathway have been identified. miR-34a, a promoter of senescence, inhibits the expression of SIRT1 by combining with the 3'UTR of SIRT1; it participates in the senescence of vascular endothelial progenitor cells 43. In addition, miR-21 is involved in the aging of vascular endothelial progenitor cells 44. miR-20 participates in the processes of cell proliferation and differentiation, which are essential for cell senescence and tissue repair and remodeling 45. The tumor suppressor miR-125a-5p participates in the development and progression of cancer by regulating cell proliferation and apoptosis 46. miR-125a-5p also inhibits cell proliferation and induces apoptosis by targeting BCL2, BCL2L12, and Mcl-1 47.

In the present study, we found that, compared with the control group, the levels of miR-125a-5p in normal smokers and COPD smokers increased. When miR-125a-5p was knocked down, the cell senescence markers p21 and p27 were reduced, and the staining of SA-β-gal positive cells was lowered, leading to inhibition of the secretion of SASP by MLE-12 cells. Further, we instilled an miR-125a-5p inhibitor into the noses of mice. In this way, we knocked down the levels of miR-125a-5p in the lung tissue of mice, and found that the degree of lung aging caused by CS exposure was reduced. Thus, miR-125a-5p is a positive regulator of cell senescence, and it participates in the senescence process of lung epithelial cells induced by CS.

To determine the target of miR-125a-5p, the bioinformatics website was used to characterize Sp1. miRNAs target various protein-coding genes by binding to the 3'UTR regions, thereby mediating intracellular effects 48. Sp1, a widely studied transcription factor, regulates the transcription of various genes related to cell proliferation and signaling pathways 49. We demonstrated that miR-125a-5p targets the 3'-UTR of Sp1 mRNA, and that its overexpression is related to the inhibition of luciferase activity. In addition, after miR-125a-5p knockdown, there was an increase in Sp1 protein levels, indicating that miR-125a-5p targets Sp1 mRNA for post-transcriptional regulation. Sirtuin is a deacetylase, and members of the sirtuin family prolong the lifespan of yeast 50. Sirtuins inhibit human aging through various signal pathways. SIRT1, a NAD^+^-dependent type III histone deacetylase, deacetylates histones (including H1, H2, H3, and H4), along with a variety of non-histone factors 51. SIRT1 inhibits HIF-1α signaling by deacetylating HIF-1α on Lys674 52. In the process of cell senescence, SIRT1 is inhibited. Therefore, the level of HIF-1α acetylation increases, which activates the transcriptional regulation of downstream target genes. Our research shows that when SIRT1 in MLE-12 cells is depleted, the acetylation level of HIF-1α is increased. Levels of SIRT1, a molecular target downstream of Sp1, are enhanced by Sp1 27, 28. We observed that overexpression of Sp1 increased the level of SIRT1, and knockdown of Sp1 decreased the level of SIRT1. CHIP experiments confirmed that Sp1 binds to the SIRT1 promoter in MLE-12 cells, a result in line with a previous study 28.

To confirm that miR-125a-5p participates in the senescence process of MLE-12 cells induced by CS through regulation of Sp1, we transfected CSE-treated MLE-12 cells with an miR-125a-5p inhibitor and Sp1 siRNA. The results showed that Sp1 knockdown reduced the inhibitory effect of the miR-125a-5p inhibitor on MLE-12 cell senescence, the expression levels of Sp1 and SIRT1 were reduced, the level of HIF-1α acetylation increased, and the cellular senescence markers p21 and p27 were up-regulated. Measurement of SA-β-gal staining and SASP secretion levels confirmed this. For mice exposed to CS in a COPD/emphysema model, knocking down miR-125a-5p reduced the emphysema caused by CS, and the lung aging index proteins and pathway proteins were inhibited, which verified our hypothesis. Thus, the results indicate that miR-125a-5p regulates SIRT1/HIF-1α through Sp1 and participates in CSE-mediated MLE-12 cell senescence and SASP factor secretion.

## Conclusion

**Figure 8:**
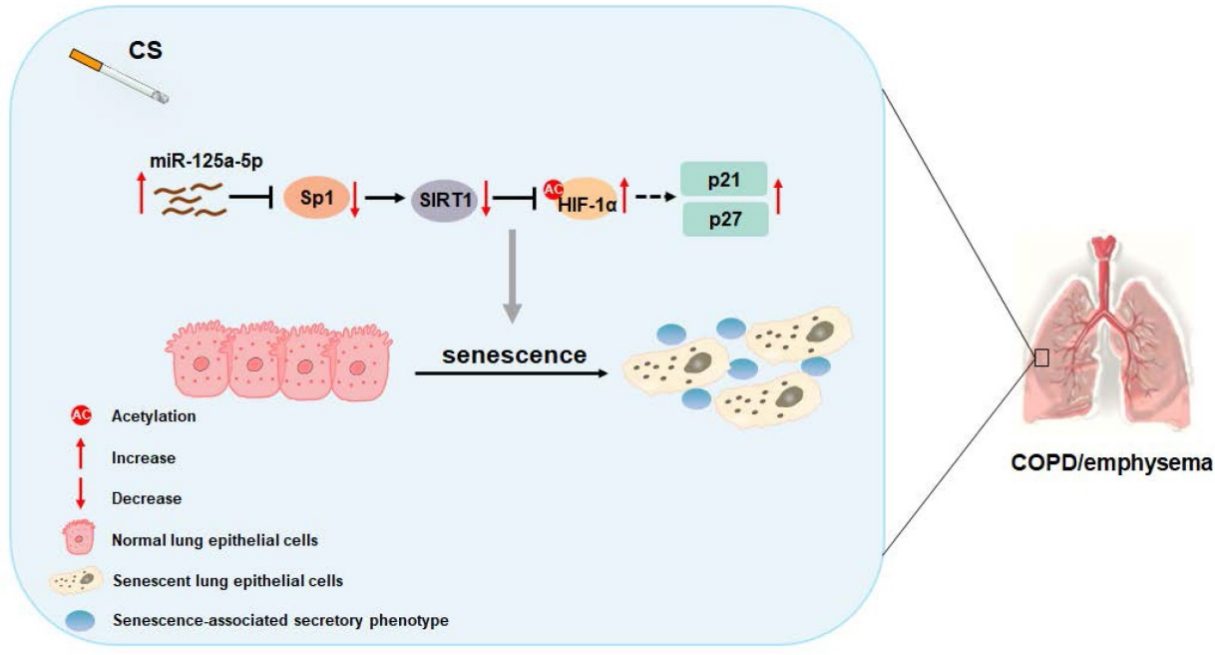
** Schematic illustration of the proposed roles of miR-125a-5p in smoking-caused senescence and COPD/emphysema.** CS exposure increases the levels of miR-125a-5p in lung epithelial cells, which reduces levels of its target protein Sp1 and levels of SIRT1. The levels of acetylated HIF-1α are elevated, leading to its activation, which enhances the transcription of p21 and p27. This process leads to senescence of lung epithelial cells and COPD/emphysema.

In sum, as summarized in Figure 8, our data indicate that, in smoking-induced COPD/emphysema, miR-125a-5p via Sp1 regulation of SIRT1/HIF-1α is involved in the senescence of lung epithelial cells. Our findings have diagnostic and therapeutic implications for COPD/emphysema.

## Supplementary Material

Supplementary figures and table.

## Abbreviations

COPD: Chronic obstructive pulmonary disease
FEV1/FVC: forced expiratory volume in one second/forced vital capacity
CSE: cigarette smoke extract
CS: cigarette smoke
SASP: senescence-associated secretory phenotype
MLE: mouse lung epithelial
SA-β-gal: senescence-related β-galactosidase
miRNA: MicroRNA
UTRs: untranslated regions
AHR: airway hyper-responsiveness
IHC: immunohistochemical
TPM: total particulate matter
BALF: bronchoalveolar lavage fluid
DDR: DNA damage response
