# Supplementary material for: Regulation of lung epithelial cell senescence in smoking-induced COPD/emphysema by microR-125a-5p via Sp1 mediation of SIRT1/HIF-1a
Source: Int J Biol Sci. 2022 Jan 1;18(2):661–74. doi: 10.7150/ijbs.65861 (PMC8741857; doi:10.7150/ijbs.65861)
Supplement: Supplementary file 1 — Supplementary figures and table. [file ijbsv18p0661s1.pdf]

Supplementary materials

Supplementary tables

Table S1. Clinical characteristics of study subjects

	Never smokers	Smokers	Smokers with COPD/emphysema
n	20	20	20
Age (years)	64±8.5	67±10.7	69±9.3
Gender (male/female)	20/0	20/0	20/0
BMI (kg/m^2)	23±3	26±4	24±4
Smoking history (pack-years)	0	27±12	36±18$^{\#}$
FEV1/FVC（%）	86.49±8.12	81.56±6.00**	60.41±5.33**$^{\#\#}$

$^{**}p<0.05$, compared with the non-smoker group, $^{\#}p<0.05$ and $^{\#\#}p<0.01$ compared with the smokers group.

Table S2. Sequences of primers used

**Gene Name**	**Sequences (5'-3')**
miR-125a-5p F	*GGGTCCCTGAGACCCTTTAAC*
miR-125a-5p R	*CAGTGCGTGTCGTGGAGT*
miR-34a-5p F	*GGGTGGCAGTGTCTTAGCT*
miR-34a-5p R	*CAGTGCGTGTCGTGGAGT*
miR-20a-5p F	*GGGTAAAGTGCTTATAGTGC*
miR-20a-5p R	*CAGTGCGTGTCGTGGAGT*
U6 F	*CGCTTCGGCAGCACATATACTAAAATTGGAAC*

U6 R	*GCTTCACGAATTTGCGTGTCATCCTTGC*
SIRT1 promoter F	*TCATTCTGACTGTGATGACGA*
SIRT1 promoter R	*CTGCCACAGTGTCATATCCAA*
TGF-β1 F	*ACTGGAGTTGTACGGCAGTG*
TGF-β1 R	*GGGGCTGATCCCGTTGATTT*
Snail F	*ATGGAGTGCCTTTGTACCCG*
Snail R	*CAGTAACCACCCTGCTGAGG*
p21 F	*TAAGGACGTCCCACTTTGCC*
p21 R	*CTGAGGATCACCCCCAGGTA*
GAPDH F	*CCCTTAAGAGGGATGCTGCC*
GAPDH R	*ACTGTGCCGTTGAATTTGCC*

Supplementary Figures and figure legends

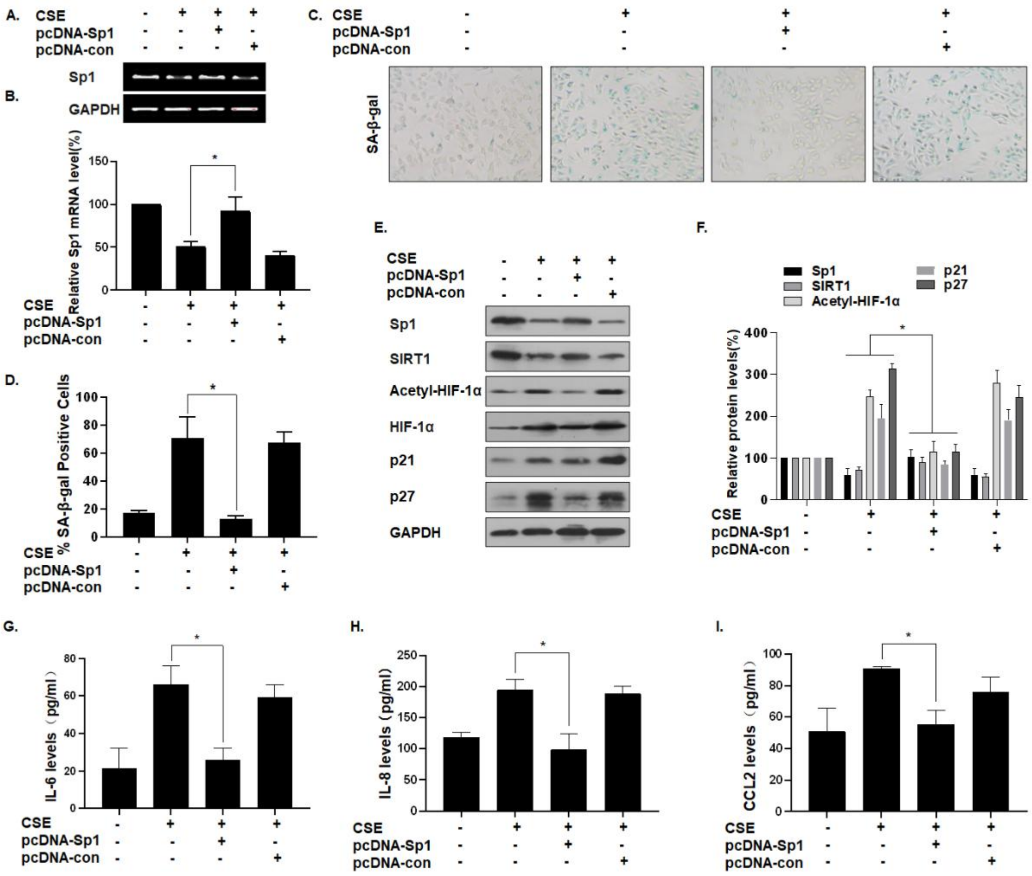

Figure S1. Sp1 is involved in CSE-mediated MLE-12 cell senescence and secretion of SASP factors.

Densities of bands were quantified by Image J software. GAPDH levels, measured in parallel, served as controls. MLE-12 cells were exposed to 0 or 8% CSE for 48 h after cells were transfected with an pcDNA-Sp1 or an pcDNA-con for 24 h. (A and B) The levels of Sp1 in MLE-12 cells were measured by RT-PCR (n = 3) and qRT-PCR (n = 3). (C) Photographs of senescence-associated β-galactosidase (SA-β-gal) staining and (D) the percentage of SA-β-gal positive cells (n = 3). (E) Western blots were performed, and (F) the relative protein levels of Sp1, SIRT1, acetyl-HIF-1α, HIF-1α, p21, and p27 in MLE-12 cells were determined (n = 3). The levels of IL-6 (G), IL-8 (H) and CCL2 (I) in MLE-12 cells were assessed by ELISA (n = 3). Values were

expressed as means ± SD. * $p < 0.05$ compared with individual CSE treatment groups.
